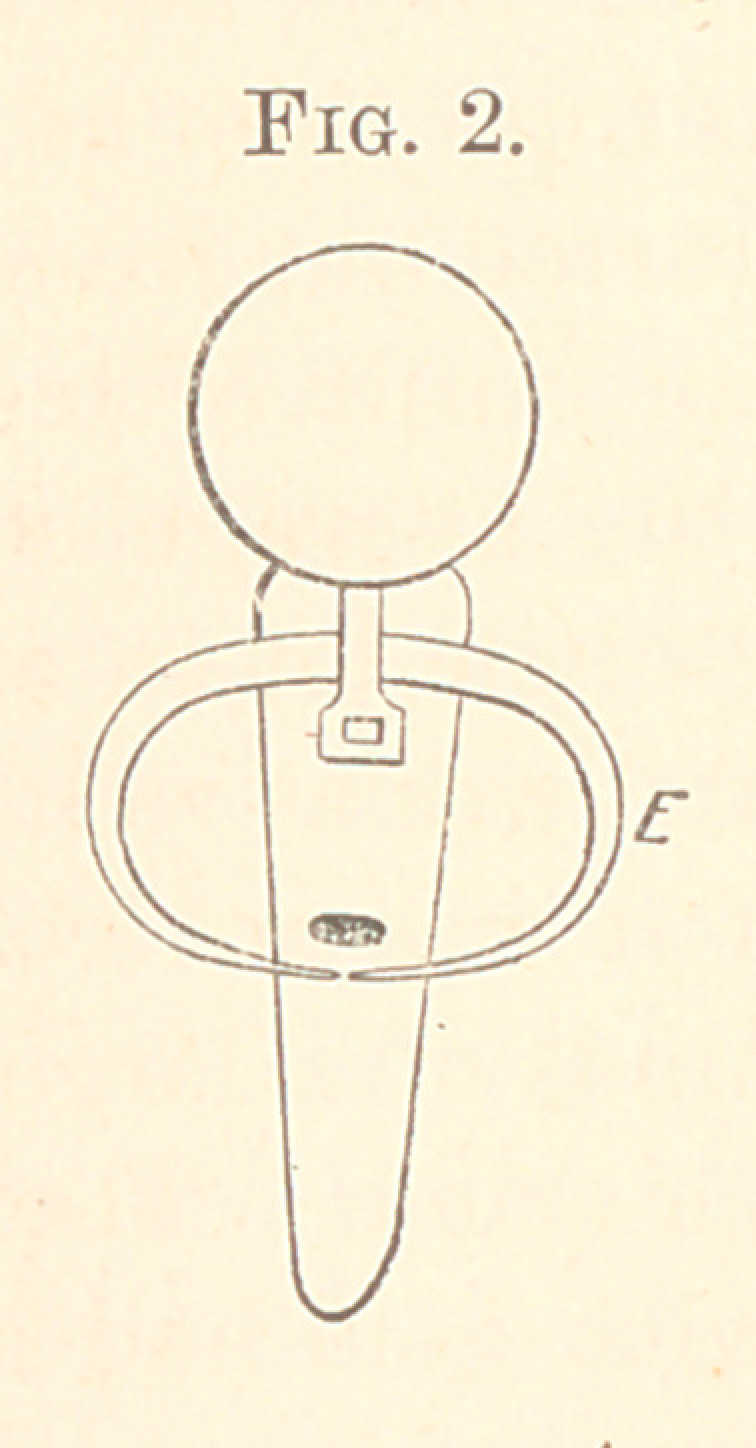# Labial Rubber-Dam Clamp

**Published:** 1892-03

**Authors:** William A. Woodward

**Affiliations:** New York


### LABIAL RUBBER-DAM CLAMP.

BY WILLIAM A. WOODWARD, D.D.S., NEW YORK.

The cuts illustrate a new device for retaining the rubber dam above the cervical margins of labial cavities. It is a modification of a clamp designed by Dr. Hatch, of San Francisco, California. Fig. 1 represents the clamp adjusted on an inferior cuspid. The gum extends very low down on the lingual surface, and quite the reverse on the labial surface; a clamp with a square grip would be useless for this case. It has three points of bearing which insure even contact; screw D is one point, the other two are at the neck of the tooth from the clamp. It will take the same position as represented in Fig. 1 on a long tooth, without the assistance of screw D,

Fig. 1.

Fig. 2.

the clamp resting on the cutting edge of the tooth, and the sides of the bow E, Fig. 2, on the adjoining teeth. The screw D is used for short teeth only, as in nineteen cases out of every twenty the clamp will retain the dam without it.

It can be so regulated that there will be no infringement on the gum, and also to secure a square grip if required.

On the surface of the lever A are three counter-sunk indentations for the point of screw B to engage.

This screw-point never slips from these indentations, which give steadiness and firmness of retention to the clamp.

From the cross-bars C a hinge-like movement is secured, change the position of its grip, and allow screw-point B to enter the different indentations. Fig. 2 shows the clamp in position, and cavity high up in cementum.

It is intended for single-root teeth, for which it has worked to perfection ; it has occasionally fitted even a molar.

It was exhibited at the November meeting, 1891, of the New York Odontological Society.

## Figures and Tables

**Fig. 1. f1:**
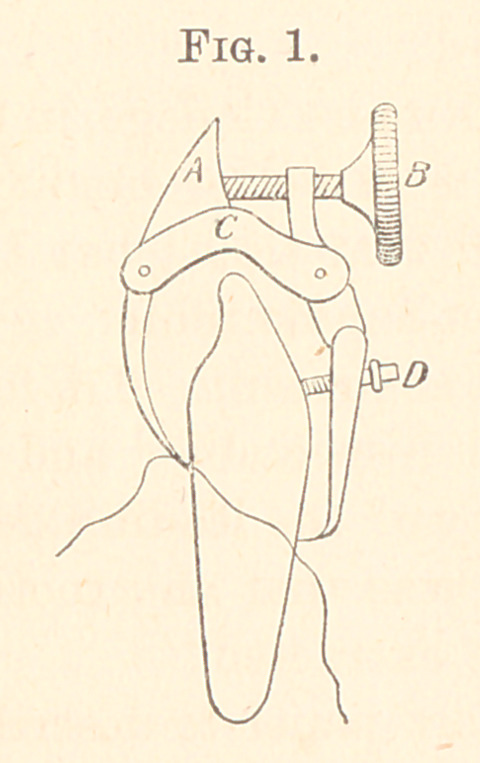


**Fig. 2. f2:**